# Curcumin assists anti-EV71 activity of IFN-α by inhibiting IFNAR1 reduction in SH-SY5Y cells

**DOI:** 10.1186/s13099-022-00481-5

**Published:** 2022-02-12

**Authors:** Yanfang Wang, Kena Dan, Xiaoling Xue, Bangtao Chen, Cheng Chen

**Affiliations:** 1 Department of Infectious Diseases, Jincheng People’s Hospital, Jincheng, 048000 China; 2 Department of Dermatology, The Third Affiliated Hospital of Chongqing Medical University, Chongqing, 401120 China; 3 Department of Hematology, The Third Affiliated Hospital of Chongqing Medical University, Chongqing, 401120 China; 4 Department of Dermatology, Chongqing University Three Gorges Hospital, Chongqing, 404100 China; 5 Department of Gastroenterology, Chongqing Key Laboratory of Translational Research for Cancer Metastasis and Individualized Treatment, Chongqing University Cancer Hospital, Chongqing, 400030 China

**Keywords:** *Enterovirus* 71(EV71), Interferon alpha receptor 1 (IFNAR1), Curcumin, Interferon-α (IFN-α), Ubiquitin–proteasome

## Abstract

**Background and aim:**

*Enterovirus* 71(EV71) can cause severe hand, foot, and mouth disease (HFMD) with brain tissue involvement. Few effective anti-EV71 drugs are presently available in clinical practice. Interferon-α (IFN-α) was ineffective while Curcumin was effective in restricting EV71 replication in non-neuronal cells. Ubiquitin–proteasome-mediated degradation of interferon-alpha receptor 1 (IFNAR1) protein contributes to IFN-α resistance. Current study aimed to determine synergistic inhibition of EV71 by Curcumin and IFN-α in human neuroblastoma SH-SY5Y cells.

**Methods:**

SH-SY5Y cells were infected with mock-/Curcumin-pre-incubated EV71 or transfected with plasmid containing interferon-stimulated response element (ISRE) or mRNA containing viral internal ribosomal entry site (IRES) following by post-treatment with Curcumin with or without IFN-α. Supernatant IFN-α/β was detected by ELISA. ISRE, IRSE, proteasome and deubiquitinating activity were measured by luciferase assay. EV71 RNA and viral protein or IFNAR1 were determined by qPCR and western blot, respectively.

**Results:**

EV71 flailed to completely block IFN-α/β production but inhibited IFN-α signal. Curcumin only slightly inhibited EV71 proliferation without modulating virus attachment and internalization. However, Curcumin addition restored IFN-α-mediated ISRE activity thus significantly inhibiting EV71 replication. Furthermore, EV71 also reduced IFNAR1 protein with proteasome-dependence in SH-SY5Y cells, which can be reversed by Curcumin addition with the evidence that it lowered proteasome activity.

**Conclusion:**

These data demonstrate that Curcumin assists anti-EV71 activity of IFN-α by inhibiting IFNAR1 reduction via ubiquitin–proteasome disruption in SH-SY5Y cells.

## Introduction

*Enterovirus* 71(EV71), one member of genus *Enterovirus* in the family *Picornaviridae*, is the major causative pathogen of severe hand, foot, and mouth disease (HFMD) characterized by brainstem encephalitis, acute flaccid paralysis, neurological pulmonary edema, cardiopulmonary dysfunction, and even death. Structurally, the icosahedral virus particle harbors a single-stranded and positive-sense RNA genome with two open reading frames flanked by a 3′-untranslated region (UTR) with a poly (A) tail and a highly structured 5′-UTR [1]. The genome encodes seven non-structural proteins (2A–2C and 3A–3D) and four structural viral proteins (VP1–VP4) by viral 5′UTR contained internal ribosomal entry site (IRES)-driven translation [2].

To date, no established antiviral treatments are applied for severe HFMD because its pathogenicity remains to be further understood. To evade the host’s antiviral innate immunity in the process of virus-host interaction, EV71 has evolved a moiety of strategies and inhibition of type I interferon (IFN-I) response by viral protease 2A and 3C is mainly involved [3]. IFN-I response refers to IFN-I production and IFN-I signaling. In IFN-I signaling, IFN-α or β activates interferon-stimulated response element (ISRE) activity by attaching to interferon alpha receptor (IFNAR) complex on cell surface. We and other teams previously proved that EV71 captures to achieve an antagonistic effect on IFN-I by reducing IFNAR1 protein in a caspase-3- or ubiquitin–proteasome-dependent manner [4, 5].

Exploration of inhibitors that reverse EV71-mediated IFN-I antagonism promises the development of new antiviral strategies. Curcumin, the primary Curcuminoid derived from the rhizome of *Curcuma longa* plant, showed anti-viral activity in vitro or in vivo at various stages of lifecycle of virus propagation [6]. Previous research has demonstrated that Curcumin can inhibit EV71 replication in non-human Vero cells, human intestinal epithelial HT29cells and rhabdomyosarcoma (RD) cells via different mechanisms [7–10]. However, it remains largely unknown whether Curcumin disrupts EV71 propagation in human neural cells as the neurotropism of EV71. In the present study, we found that EV71 proliferation in SH-SY5Y cells was only slightly inhibited by Curcumin at a safe dose, however, Curcumin significantly assisted anti-EV71 activity of IFN-α via IFNAR1 restoration by disrupting 20S proteasome activities.

## Materials and methods

### Cell culture and virus preparation

Human RD cells and human neuroblastoma SH-SY5Y cells were maintained in Dulbecco’s modified Eagle’s medium (DMEM, Gibco) containing 10% fetal bovine serum (FBS, Hyclone) with 100 U/mL penicillin and 100 μg/mL streptomycin. Neurotropic strain EV71 (Xiangyang-Hubei-09, GenBank accession no. JN230523.1) was propagated and titrated in RD cells and stored at − 80 °C until use.

### Cell infection and stimulation

SH-SY5Y cells were infected with EV71 at the multiplicity of infection (MOI) of 5. Observation of cell morphology was performed with microscope. Different concentrations of double-stranded alternating copolymer Poly(dAT:dAT) (#P0883, Sigma-Aldrich), Curcumin (#S1848, Selleck) and human IFN-α (#ab73124, Abcam) were used at the time points indicated in the figure legends. Cytotoxicity of Curcumin with and without IFN-α was measured by the MTT assay according to related manufacturer’s instructions and what described previously [11], each treatment replicates three times. The protein level of IFN in cell supernatants was detected with human IFN-α ELISA kit (#EHC144a, neobioscience) and human IFN-β ELISA kit (#EHC026b, neobioscience) following manufacturer’s instructions and all the plates were read by the I Mark™ Micro plate Reader (BIO-RAD).

### Plasmid, transfection and luciferase assay

The construction of bi-cistronic reporter plasmid containing Cap-Rluc-*v*IRES-Fluc and its transcription in vitro were done as previously described [12]. Reporter plasmids *p*GM-ISRE-RLuc and *p*GM-Fluc were purchased from Yeasen Co., Ltd. Transfection experiments were performed using Lipofectamine^2000^ reagent (Life Technologies) according to the manufacturer’s instruction. As indicated in figure legends, cells seeded in 96-well plates were transfected with the bi-reporter mRNAs (100 ng/well) followed by Curcumin treatment to monitor the effect of Curcumin on the IRES-dependent translation efficiency, cells seeded in 96-well plates were co-transfected with plasmids *p*GM-ISRE-RLuc (100 ng/well) and *p*GM-Fluc (20 ng/well) followed by IFN-α with or without Curcumin treatment to monitor ISRE activity. Cells were then lysed for luciferase assay using the Luciferase Assay System Kit (Promega) according to manufacturer’s instructions.

In addition, fluorogenic substrates SLLVY-AMC and ubiquitin-AMC were used to determine the chymotrypsin-like activity of the 20S proteasome and deubiquitinating activities, respectively, as Si et al. and Qin et al. reported [8, 13]. Briefly, fresh cytoplasmic proteins were extracted with RIPA buffer from treated or untreated SH-SY5Y cells in absence of protease inhibitor, and the concentrations of the proteins were determined; then 20 μg of cytoplasmic protein was added to an assay buffer [20 mM Tris–HCl (pH 8.0), 1 mM ATP, and 2 mM MgCl_2_] in the presence of 75 μM SLLVY-AMC substrate to a final volume of 100 μL in a 96-well microplate. In parallel, the SH-SY5Y cells were dissolved in suspension buffer [20 mM HEPES-K(pH 7.5), 10 mM KCl, 5 mM MgCl_2_, 0.5 mM dithiothreitol, 0.5 mM EDTA, 0.1 mM phenylmethylsulfonyl fluoride, 0.5% NP-40, DNase I (50 μg/mL), 4% glycerol] and the supernatant was collected for quantification of protein concentrations; then 10 μg of cytoplasmic protein was added to an assay buffer [50 mM HEPES–NaOH(pH 7.8), 0.5 mM EDTA, 1 mM dithiothreitol, 0.1 mg/mL ovalbumin] in the presence of 1 μM ubiquitin-AMC substrate to a final volume of 100 μL in a 96-well microplate. The microplates were then incubated at 30 °C for 1 h, and the fluorescence product SLLVY-AMC and ubiquitin-AMC were determined by a microplate reader at an emission wavelength of 465 nm.

All luciferase reporter and fluorogenic substrate AMC assays were carried out at least three times.

### Virus attachment and internalization

5 MOI of EV71 were pre-incubated with Curcumin at 37 °C for 2 h, then the virus were used to infect cells with binding buffer on ice. 1 h later, the cells were washed (attachment assessment) or/and cultured at 37 °C for another 1 h and then treated with trypsin (internalization assessment). Viral RNA was extracted by commercial kit and EV71 RNA was determined by qPCR performed as previously described [14].

### Western blot and antibodies

The SH-SY5Y whole-cell lysates were prepared by lysing with RIPA buffer and western blot was performed as previously described [4, 12]. Anti-EV71 VP1 (#PAB7631-D01P) and anti- IFNAR1 (#ab45172) were obtained from Abnova and Abcam, respectively. Anti-IFNAR2 (#A1769) was purchased from ABclonal. Anti-β-actin (#BE0021-1000), anti-rabbit (#BE0103-100) and anti-mouse (#BE0108-100) secondary antibody conjugated with horseradish peroxidase were purchased from EASYBIO. Specific bands were visualized with enhanced chemiluminescent substrate (ECL) and density of visualized bands was conducted using Quantity One software. Each immunoblot assay was carried out at least three times and one of them was presented.

### Statistical analyses

Data were compiled in excel and analyzed using GraphPad Prism software (GraphPad Software Inc., La Jolla, CA), the results were expressed as the mean ± standard deviation (SD) obtained from the experiments repeated at least three times. Statistical analysis was performed using *Student’s* t-test among two groups. A *P* value < 0.05 level was accepted as the cutoff for statistical significance.

## Results

### EV71 blocks IFN-α signal in SH-SY5Y cells

**Fig. 1:**
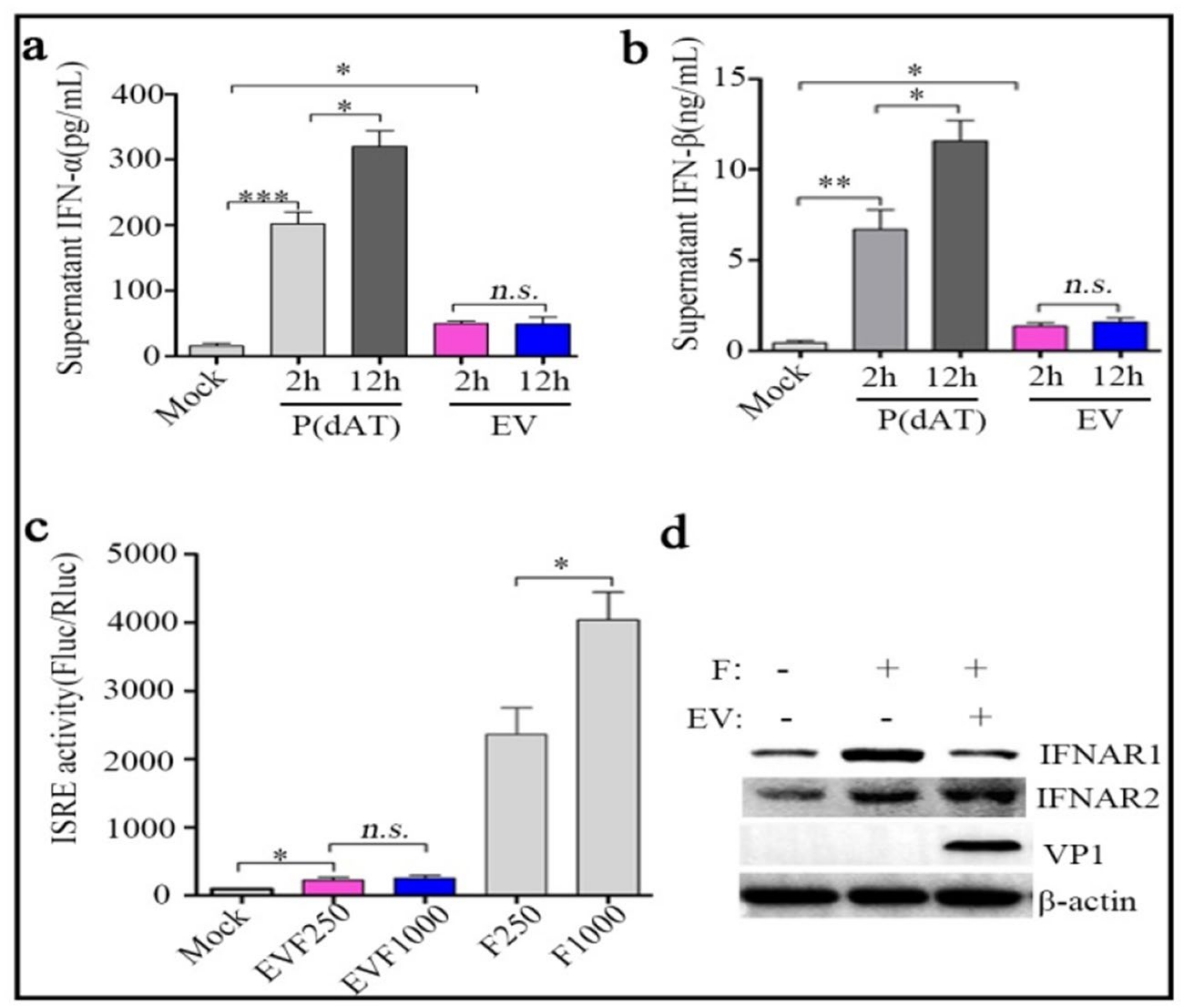
EV71 blocks IFN-α signal in SH-SY5Y cells. SH-SY5Y cells were seeded in 6-plates and infected with neurotropic strain EV71 (Xiangyang-Hubei-09, GenBank Accession No. JN230523.1) at an MOI of 5 or treated with 2 μg/well Poly(dAT:dAT). 2 h and 12 h later, cellular supernatants were used to determined protein levels of supernatant IFN-α (**a**) and IFN-β (**b**) by ELISA following manufacturer’s instructions. **c** SH-SY5Y cells were firstly co-transfected with *p*-ISRE-FLuc (100 ng/well, 96-well plate) and *p*-RLuc (20 ng/well, 96-well plate) for 24 h, then the cells were infected with mock or EV71 for 10 h. After that, the cells were treated with 250 or 1000 IU/mL IFN-α for another 2 h. Intensity of FLuc and RLuc were measured as Materials and Methods described and Fluc/Rluc indicates ISRE activity. **d** SH-SY5Y cells seeded in 6-plates were treated in parallel as shown in **c**, total cellular protein were extracted and used for the detection of IFNAR1,IFNAR2, VP1 or β-actin protein by western blot, β-actin were used as loading control. All results indicate the mean ± SD of three independent experiments. Statistical analysis was performed using *Student’s* t-test, *P < 0.05, ** *P* < 0.01, *** *P* < 0.001, *n.s. P* > 0.05. EV, EV71; P(dAT), Poly(dAT:dAT); F, IFN-α

To determine whether neurotropic strain EV71 infection could induce IFN-I production in neuroblastoma SH-SY5Y cells, the cells were infected with EV71 at an MOI of 5 for 2 and 12 h. ELISA analysis revealed that virus caused moderate increase of supernatant IFN-α and IFN-β at hour 2 and 12 after infection (Fig. 1a and b). In contrast to that, IFN-α and IFN-β were remarkably increased in time-dependent manner in the cells treated with Poly (dAT:dAT), the ligand of retinoic acid inducible gene I(RIG-I). To further examine the effect of EV71 on IFN-I signal, ISRE activity and IFNAR1 protein levels in mock- or EV71-infected SH-SY5Y cells with IFN-α (250 or 1000 IU/mL) post-treatment were assessed. Our luciferase assay showed that the IFN-α-primed ISRE activities were significantly suppressed by EV71 infection and western blot demonstrated that IFN-α treatment increased IFNAR1 protein in mock-infected SH-SY5Y cells, which was absent in EV71-infected SH-SY5Y cells; however, addition of IFN-α could increase IFNAR1 protein in SH-SY5Y cells with or without EV71 infection (Fig. 1c and d). These data confirm the pivotal contribution role of IFNAR1 protein reduction and subsequent ISRE inhibition in EV71-mediated IFN-α unresponsiveness in SH-SY5Y cells.

### Curcumin slightly inhibits EV71 proliferation

**Fig. 2:**
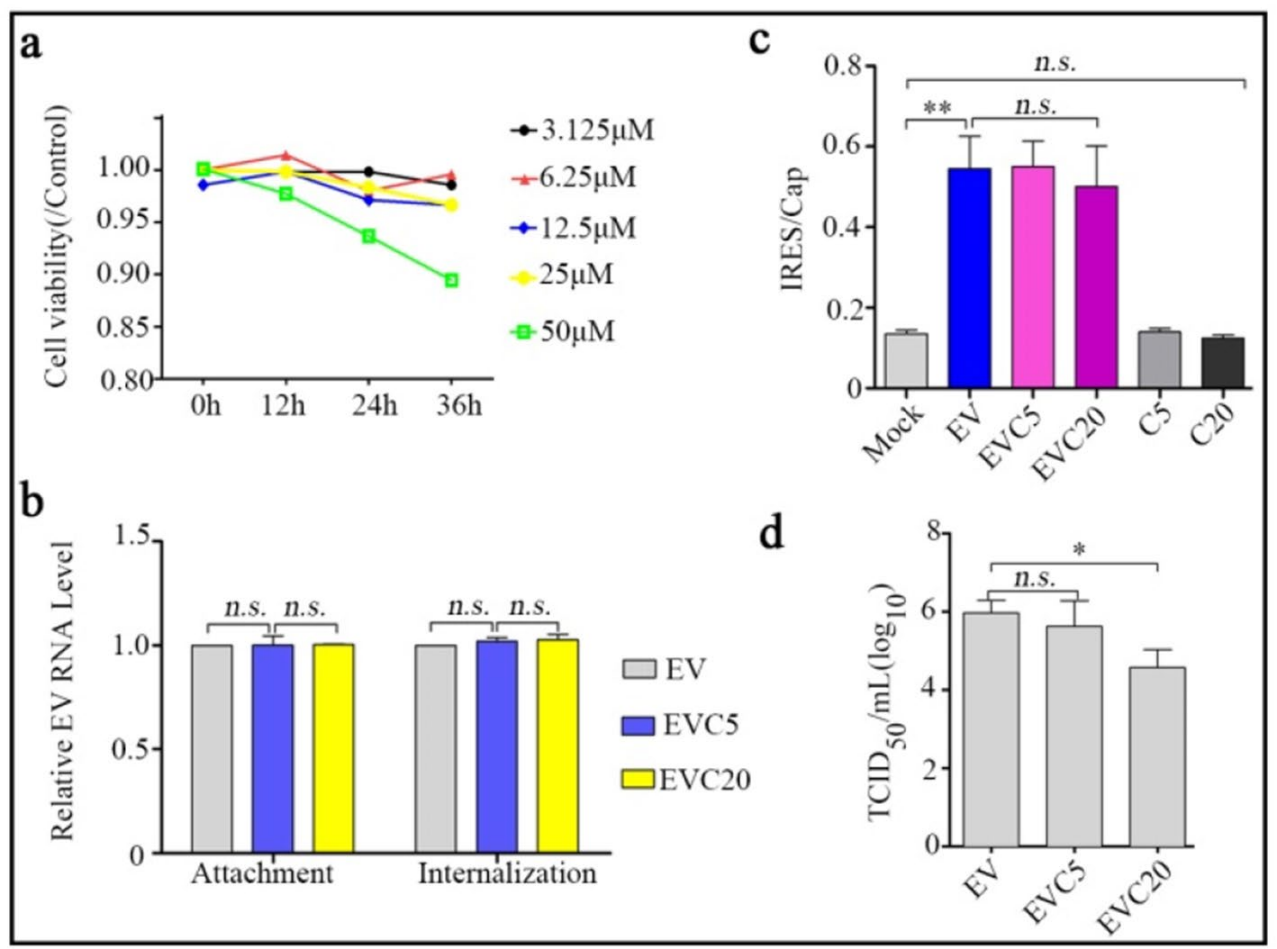
Curcumin slightly inhibits EV71 proliferation. SH-SY5Y cells were treated with 3.125 to 50 μM Curcumin for 0 to 36 h followed by cell viability assessment using MTT (**a**). 5 MOI of EV71 were pre-incubated with 5 μM or 20 μM Curcumin at 37 °C for 2 h, then the virus were used to infect SH-SY5Y cells with binding buffer on ice. 1 h later, the cells were washed (attachment assessment) or/and cultured at 37 °C for another 1 h and then treated with trypsin (internalization assessment). Viral RNA was extracted by commercial kit and EV71 RNA was determined by RT-qPCR (**b**). SH-SY5Y cells were pre-transfected with Cap-Rluc-*v*IRES-Fluc mRNA (100 ng/well, 96-well plate). 4 h later, the cells were infected with mock or EV71 for 2 h and then the cells were treated with 5 or 20 μM Curcumin for 10 h. Intensity of FLuc and RLuc were measured as Materials and Methods described and Fluc/Rluc indicates IRES activity (**c**). Progeny viruses in supernatant from Fig. 1c were titrated using RD cells and the results presented as log_10_ TCID_50_/mL (**d**). All the results indicate the mean ± SD of three independent experiments. Statistical analysis was performed using *Student’s* t-test, *P < 0.05, ** *P* < 0.01, *n.s. P* > 0.05. EV, EV71; C, Curcumin

Curcumin was reported to inhibit EV71 in non-neural cells via multiple pathways, however, the effect of Curcumin on EV71 life-cycle and IFN-α signal in neural cells remains unknown. Herein, cytotoxicity of Curcumin to SH-SY5Y cells was firstly determined and results found that Curcumin at less than 25 μM was not toxic to the cells (Fig. 2a). By analysis with RT-qPCR, comparable levels of EV71 RNA on cell surface and that entering the cell were detected in 5 or 20 μM Curcumin co-incubation group in comparison with mock co-incubation group (Fig. 2b). Furthermore, 5 or 20 μ Curcumin also failed to suppress EV71- or mock-stimulated IRES activity (Fig. 2c). However, as Fig. 2d showed, intracellular viral titer was slightly decreased by post-treatment with 20 μM Curcumin (*P* < 0.05). These results imply that Curcumin post-treatment could slightly inhibit EV71 proliferation in SH-SY5Y cells.

### Combination of curcumin and IFN-α significantly inhibits EV71 replication

**Fig. 3:**
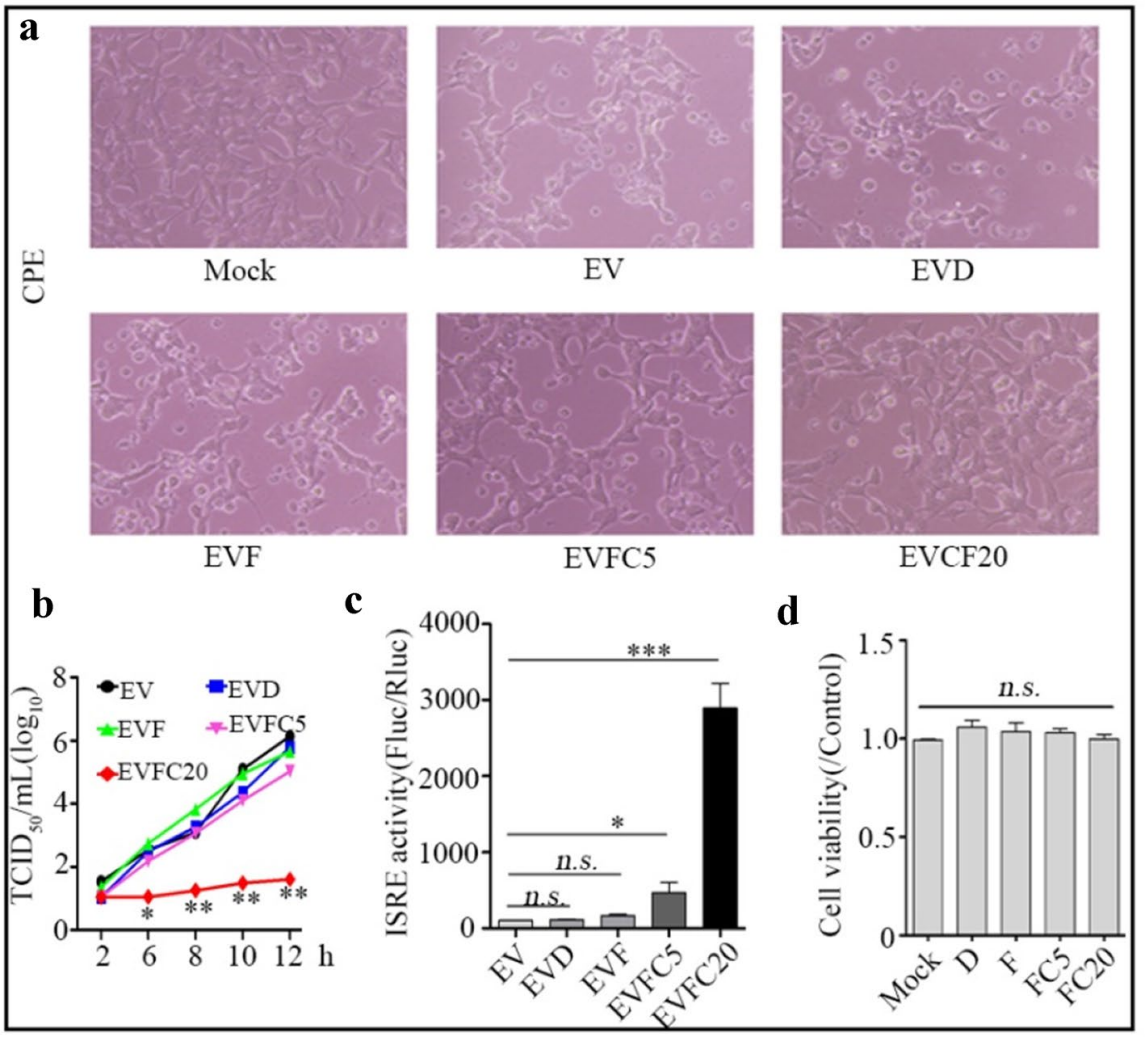
Combination of Curcumin and IFN-α significantly inhibits EV71 replication. **a** SH-SY5Y cells were infected with EV71 at an MOI of 5. 2 h later, the cells were post-treated only with DMSO or IFN-α (250 IU/mL) or the combination of IFN-α with 5 or 20 μM Curcumin. At 12 h post infection, photomicrographs were taken (original magnification, 100×). **b** SH-SY5Y cells were infected with EV71 at an MOI of 5. 2 h later, the cells were post-treated only with DMSO or IFN-α (250 IU/mL) or the combination of IFN-α with 5 or 20 μM Curcumin. At 2,6,8,10,12 h post infection, intracellular progeny viruses were titrated using RD cells and the results presented as log_10_ TCID_50_/mL. **c** SH-SY5Y cells were co-transfected with *p*-ISRE-FLuc (100 ng/well, 96-well plate) and *p*-RLuc (20 ng/well, 96-well plate) for 24 h, then the cells were treated in parallel as shown in **a**, intensity of FLuc and RLuc was measured as Materials and Methods described and Fluc/Rluc indicates ISRE activity. **d** SH-SY5Y cells were treated with DMSO or IFN-α (250 IU/mL) or the combination of IFN-α with 5 or 20 μM Curcumin for 12 h followed by cell viability assessment using MTT. All the results indicate the mean ± SD of three independent experiments. Statistical analysis was performed using *Student’s* t-test, *P < 0.05, ** *P* < 0.01, *** *P* < 0.001, *n.s. P* > 0.05. EV, EV71; D, DMSO; F, IFN-α; C, Curcumin

To assess the antiviral properties of Curcumin in combination with IFN-α and prospects in medicinal potential, we further tried to determine the effect of combination of Curcumin and IFN-α post-treatment on EV71 proliferation in vitro Compared to virus-infected cells with 250 IU/mL IFN-α challenge, co-administration of 20 μM Curcumin dramatically alleviated the occurrence of cytopathic effect (CPE) of SH-SY5Y cells induced by EV71 infection (Fig. 3a). Accordingly, in comparison with IFN-α mono-treatment, combination of IFN-α and 20 μM Curcumin significantly decreased intracellular viral titers at 6 to 12 h post infection (Fig. 3b). In parallel, 5 μM and 20 μM Curcumin could moderately and significantly restored IFN-α-induced ISRE activity in virus infected cells, respectively (Fig. 3c). Notably, no obvious cytotoxicity of 250 IU/mL IFN-α in combination with 5 μM or 20 μM Curcumin to SH-SY5Y cells was observed (Fig. 3d). Collectively, these results indicate Curcumin's assistance to anti-EV71 activity of IFN-α.

### Curcumin inhibit IFNAR1 reduction by disrupting 20S proteasome activities

**Fig. 4:**
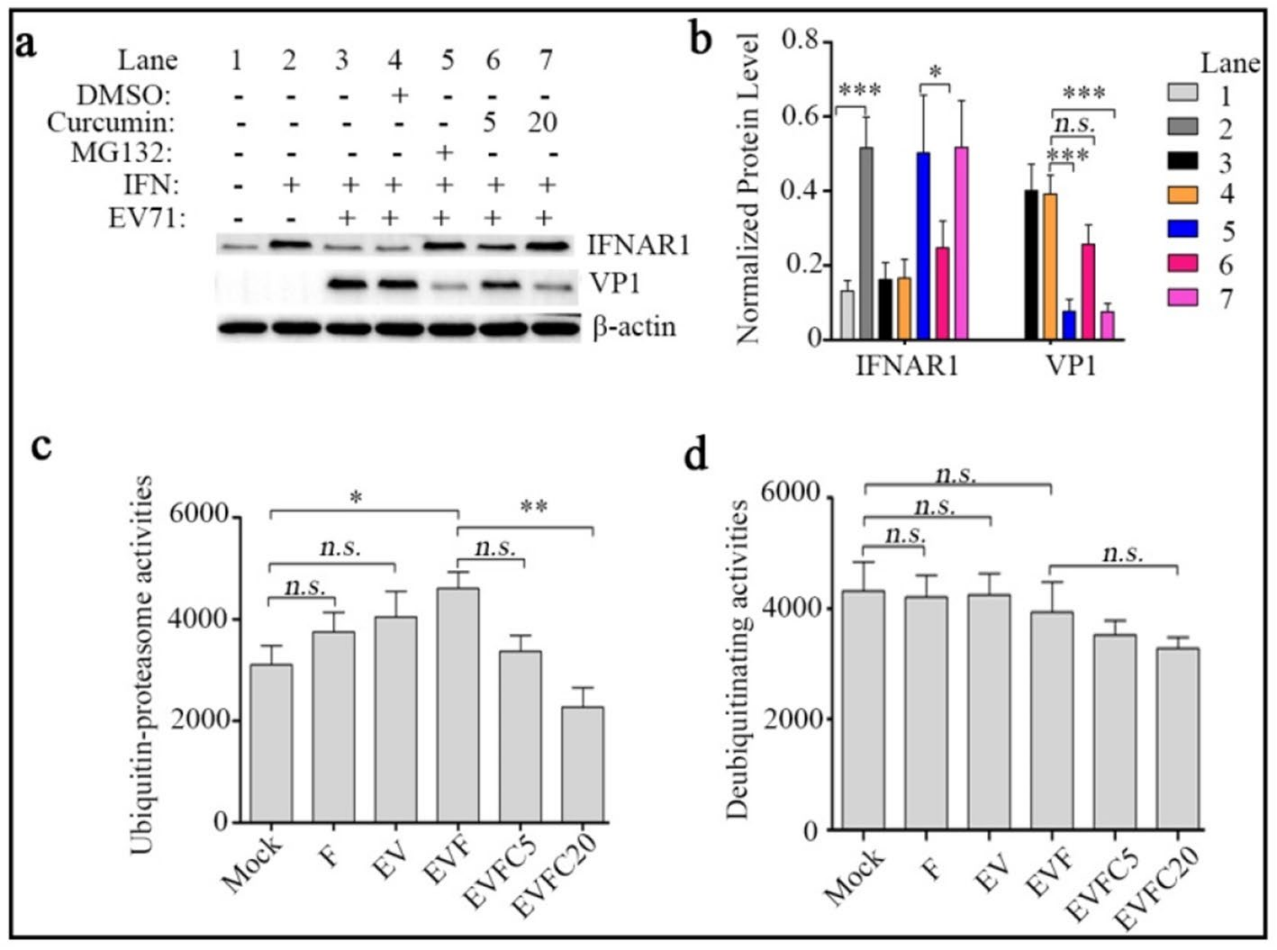
Curcumin inhibit IFNAR1 reduction by disrupting 20S proteasome activities. **a** SH-SY5Y cells were infected with EV71 at an MOI of 5. 2 h later, the cells were post-treated with IFN-α (250 IU/mL) without or with DMSO or MG132 (5 μM) or Curcumin (5 or 20 μM) as the figure outlined. 10 h later, total cellular protein were extracted and used for the detection of IFNAR1, VP1 and β-actin (loading control) protein by western blot. **b** Densitometric analysis of the western blot results in **a** was conducted using Quantity One software. IFNAR1 and VP1 expression levels were normalized by β-actin expression level. SH-SY5Y cells were treated in parallel as shown in **a**, cell lysates were prepared, proteasomes (**c**) and deubiquitinating (**d**) activities were measured using a fluorogenic substrate SLLVY-AMC and ubiquitin-AMC as described in Materials and Methods, respectively. All the results indicate the mean ± SD of three independent experiments. Statistical analysis was performed using *Student’s* t-test, *P < 0.05, ** *P* < 0.01, *** *P* < 0.001, *n.s. P* > 0.05. EV, EV71; F, IFN-α; C, Curcumin

We and other teams previously proved that many viruses could promote ubiquitin–proteasome-mediated degradation of IFNAR1 protein in IFN-independent manner in non-neural cells. Here, in SH-SY5Y cells, Fig. 4a and b Lane 5 showed that IFNAR1 protein was restored by proteasome specific inhibitor MG132 and thus facilitated IFN-α-induced inhibition of VP1 synthesis, which suggests that 20S proteasome is hijacked by EV71 during IFNAR1 reduction. Consistent with MG132 treatment group, 20 μM Curcumin displayed similar effect on protein levels of IFNAR1 and VP1 (Fig. 4a and b Lane 7), implying possible disruption in ubiquitin–proteasome activity upon Curcumin challenge. To test the hypothesis, we further measured the in vitro activity of proteasomes and deubiquitin enzymes in EV71-infected SH-SY5Y cells with IFN-α and/or Curcumin post-treatment. We observed that the activity of proteasomes was slightly increased by EV71 infection or IFN-α mono treatment (*P* > 0.05 for all variables) and significantly increased by EV71 + IFN-α (*P* < 0.05), while it was remarkably reduced by addition of 20 μM Curcumin (*P* < 0.01). On the other hand, IFN-α mono treatment, EV71 infection or the combination did not change deubiquitinating activity (*P* > 0.05 for all variables), and 20 μM Curcumin caused an moderate decrease but no significance was observed (Fig. 4c and d). The above data demonstrate that Curcumin-mediated IFNAR1 restoration is involved in its assistance to anti-EV71 activity of IFN-α via disrupting ubiquitin–proteasome activity.

## Discussion

Clinically, IFN-I resistance and side effects caused by high-dose of IFN-I are culprits for limited application of IFN-I. Much works are done to identify a potential combinational strategy for promoting IFN-I application with fewer clinical side effects. Curcumin, with anti-cancer, antioxidant, anti-microbial and anti-viral properties, was reported having synergistic effect with IFN-I in non-virus related disease. In patients with multiple sclerosis, Curcumin adds to efficacy of IFN-β on radiological signs of inflammation [15].Curcumin was proved to synergistically cooperate with IFN-β to inhibit migration and induce apoptosis of MCF-7 human breast cancer cells by up-regulation of genes associated with retinoid-IFN-induced mortality 19 (GRIM19) through signal transducer and activator of transcription (STAT) 3-dependent and STAT3-independent pathways [16]. Lee et al. found that activations of NF-kappa B and COX-2 contribute to IFN-α resistance in human A549 non-small cell lung cancer cells, but that can be reversed by addition of Curcumin [17, 18].

Replication of a wide-range of viruses can be inhibited by Curcumin. However, the antiviral potency of Curcumin against EV71 seems to appear at one or more stages and the effect of Curcumin mono-treatment on EV71 proliferation varies among studies depending on virus strain and model cells used. Different from what observed in enveloped viruses, pre-incubation of EV71 with Curcumin failed to reduce viral infectivity to Vero cells by assessment of plaque formation [10]. Without modulating virus attachment or viral IRES activity, 10 μM Curcumin can significantly reduce Strain Tainan/4643 EV71 proliferation and increase host cell viability in HT29cells by inhibiting phosphorylation of protein kinase C delta [7]. Ubiquitin–proteasome system was proved to be hijacked favoring viral replication; Qin et al. demonstrated that Strain BrCr EV71-mediated increased activity of proteasomes in Vero cells was inhibited by 20 and 40 μM Curcumin thus suppressing viral replication [8]. EV71-mediated cleavage of eukaryotic translation initiation factor 4GI (eIF4GI) was well established to inhibit host protein cap-dependent synthesis while encourage viral protein IRES-dependent translation. Lin et al. showed that Curcumin-derived carbon quantum dot (Cur-CQD) formulations with improved solubility in water lowered Strain 4643/MP4 EV71 viral proteins translation by reducing eIF4GI cleavage in RD cells and in ICR mice [9]. In current study with neurotropic strain EV71 (Xiangyang-Hubei-09) and neuronal SH-SY5Y cells used, pre-incubation of EV71 with 5 or 20 μM Curcumin didn’t alter virus attachment and internalization, Curcumin post-treatment caused no alteration in viral IRES activity while slightly inhibited intracellular viral titers. The inconsistent results may partly be owing to cell types, virus strain and the relatively lower dose used as only the dose less than 25 μM is non-toxic to SH-SY5Y cells when co-cultured for 24 h in vitro [11, 19, 20]. Our results that Curcumin slightly inhibited EV71 proliferation in neural cells indicating the limited application of Curcumin mono-treatment for curing EV71-induced severe HFMD.

Attempts to suppress virus in vitro by combination treatment of Curcumin and IFN-α or β are lacking because much works have demonstrated significant inhibition of virus by Curcumin alone [6]. However, 20 μM Curcumin alone only slightly inhibited neurotropic strain EV71 proliferation in SH-SY5Y cells in our study, which prompted us to try to adopt combination therapy strategy to combat EV71 proliferation and explore possible mechanisms. Here, we showed that combination of 20 μM Curcumin and 250 IU/mL IFN-α could significantly inhibit EV71 replication with the evidence from CPE, intracellular viral titers and ISRE activity. Moreover, growth inhibition of SH-SY5Y cells was not caused by the combination of IFN-α and Curcumin at the indicated concentrations.

Two issues are worthy of in-depth discussion in IFN-I-mediated host antiviral immunity, whether endogenous IFN-I is produced upon virus infection and whether IFN-I can effectively exert its antiviral effect. Partly consistent with what observed in RD cells and other neural cells [5, 21], we found that EV71 infection induced a moderate expression of IFN-I, indicating that IFN-I signal corruption may be the main strategy for EV71 to evade host antiviral immunity. The ‘‘portal’’ molecule IFNAR1 protein directly binding to IFN-I bears the most importance among the IFN-I signal pathway. However, downregulation of IFNAR1 protein is usually observed in the condition of IFN-I-treated cells or virus-infected cells, thus compromising the antiviral activity of IFN-I in clinical practice. Lu et al. and we previously presented the evidence that EV71 2A^pro^ targets IFNAR1 for reduction in absence of IFN-α in RD cells and HEK293T cells. Here, we added the evidence that neurotropic strain EV71 also caused IFNAR1 reduction in SH-SY5Y cells dependent on host ubiquitin-protease activity, which is roughly consistent with action of IFN-I-mediated IFNAR1degradation. Ubiquitin-protease system is also vital for enteroviruses to escape the host's innate immune response [22–24].However, several studies imply that interference in ubiquitin-protease system contributes to Curcumin-mediated enterovirus inhibition [8, 13]. Our current study further validated that Curcumin disrupted 20S proteasome activities in SH-SY5Y cells thus inhibited EV71-mediated IFNAR1 reduction and assisted anti-EV71 activity of IFN-α. It is well known that down-regulation of membrane receptors via ubiquitin-protease system requires sequential events including receptor phosphorylation, ubiquitination, endocytosis, dephosphorylation, deubiquitination and sorting to proteasome. In current study, we tried to determine ubiquitination level of IFNAR1 in different conditions and found similar low levels of IFNAR1 ubiquitination upon IFN-α or EV71 challenges, whereas that was upregulated by the addition of 20 μM Curcumin (data not shown) accompanied by upregulation of IFNAR1, which may imply that Curcumin does not affect ubiquitin ligase activity. Ubiquitination and deubiquitination of IFNAR1 are dynamically regulated by a raft of factors and more studies are expected to elucidate the exact actions.

Several limitations may confine the conclusion of present study. For one thing, experiment in vivo determines the effect and safety of oral or injected Curcumin on EV71 in neural cells is lacking. For another, the specific mechanism by which curcumin interferes with 20s proteasome remained unknown as with other reports. Moreover, whether Curcumin-induced inhibition of EV71 also resulting from interference in ubiquitin-protease-mediated downstream signal protein of IFNAR1 remains unclear. In spite of this, our data still demonstrate that Curcumin facilitates anti-EV71 activity of IFN-α by restoring IFNAR1 protein via proteasome disruption in SH-SY5Y cells.

## Conclusion

In summary, this study at least demonstrates that Curcumin alone showed limited antiviral effect against EV71 replication in SH-SY5Y cells, however, it indeed assist anti-EV71 activity of IFN-α by inhibiting ubiquitin–proteasome -mediated reduction in IFNAR1 protein.

## Data Availability

All data involved in this study is available upon reasonable request made to the corresponding author.

## Abbreviations

EV71: *Enterovirus* 71
HFMD: Hand, foot, and mouth disease
IFN-α: Interferon-α
IFNAR1: Interferon-alpha receptor 1
IRES: Internal ribosomal entry site
UTR: Untranslated region
VP: Viral proteins
IFN-I: Type I interferon
RD: Rhabdomyosarcoma
GRIM19: Genes associated with retinoid-IFN-induced mortality 19
STAT: Signal transducer and activator of transcription
eIF4GI: Eukaryotic translation initiation factor 4GI
Cur-CQD: Curcumin-derived carbon quantum dot
ISRE: Interferon-stimulated response element
CPE: Cytopathic effect
MOI: Multiplicity of infection
